# Immunopathogenic Role of Herpes Simplex Virus in Behçet's Disease

**DOI:** 10.1155/2013/638273

**Published:** 2013-11-24

**Authors:** Do Young Kim, Suhyun Cho, Min Ju Choi, Seonghyang Sohn, Eun-So Lee, Dongsik Bang

**Affiliations:** ^1^Department of Dermatology and Cutaneous Biology Research Institute, Yonsei University College of Medicine, 50 Yonsei-ro, Seodaemun-gu, Seoul 120-752, Republic of Korea; ^2^Department of Microbiology, Ajou University School of Medicine, Suwon 443-749, Republic of Korea; ^3^Department of Dermatology, Ajou University School of Medicine, Suwon 443-749, Republic of Korea

## Abstract

The role of viral infections, such as herpes simplex virus (HSV) infection, in the pathogenesis of Behçet's disease (BD) has been investigated for many years. HSV has been detected in peripheral blood leukocytes, saliva, and genital ulcers of patients with BD. Various cell adhesion molecules on cultured endothelial cells have been induced by HSV in a TNF-**α** dependent manner. In addition, a BD-like animal model was developed by inoculating ICR mouse earlobes with HSV, and antiviral treatment was effective in improving BD-like symptoms in this model. Still, there are several incompletely characterized proteins that possess antiviral properties and are being investigated as mediators of viral infection-related chronic inflammatory reactions. Although the role of HSV in the pathogenesis of BD remains to be fully established, recent research findings regarding HSV in BD have expanded our understanding of the disease and will hopefully lead to the development of more effective therapeutic agents in the near future.

## 1. Introduction: Historical Background

The role of viral infection in the pathogenesis of Behçet's disease (BD) was first suggested by Hûlusi Behçet, a Turkish dermatologist, in 1937 [1]. Early publications reported isolating virus from the ocular fluid, eye, and brain of patients with BD, but these findings were not initially confirmed by others [2–4]. With more recent advances in virology and immunology, DNA has been isolated in BD patients from various types of viruses, including herpes simplex virus (HSV), varicella zoster virus, cytomegalovirus, Epstein-Barr virus, human herpes virus 6 and 7, hepatitis virus, human immunodeficiency virus, and parvovirus B19 [5, 6]. Among these viruses, HSV is the leading candidate for playing a potentially key role in the pathogenesis of BD. *In situ* DNA-RNA hybridization techniques have demonstrated the presence of part of the HSV-1 genome in peripheral blood mononuclear cells of patients with BD [7]. Polymerase chain reaction (PCR) studies have confirmed the presence of a 211-base pair (bp) HSV-1 DNA fragment in the peripheral blood leukocytes of patients with BD [8] and demonstrated significantly greater quantities of HSV-1 DNA in the saliva, intestinal ulcers, and genital ulcers in BD patients than controls [9]. In addition, a BD-like animal model was developed by inoculating ICR mice with HSV [10, 11] and antiviral treatment was effective in improving BD-like symptoms in 40% of famciclovir treated BD mice [12].

Despite the aforementioned observations, the role of HSV in the pathogenesis of BD has not been firmly established, and the function of innate immunity and immunization treatment options remain to be elucidated. This review will discuss the current state of our knowledge regarding the role of HSV in BD and explore the possible future implications of this knowledge for the diagnosis and treatment of the disease.

## 2. Clinical Evidence Supporting the Role of HSV Infection and Detection of HSV in the Mucocutaneous Lesions in Behçet's Disease

BD is a recurrent, multisystemic inflammatory disease typically characterized by recurring oral aphthous ulcers, genital ulcers, ocular lesions, and cutaneous lesions and occasional articular, urogenital, vascular, gastrointestinal, and neurological involvement [13]. Oral ulcerations, the most common clinical manifestation of BD, include three patterns: minor ulcers, major ulcers, or herpetiform ulcers. The least common variety of oral aphthosis is herpetiform ulceration, which consists of numerous (up to 100) 2-3 mm lesions distributed throughout the oral cavity [14]. In common herpetic ulcers caused by HSV, the viral blisters quickly rupture, resulting in multiple small ulcers that often coalesce to form larger irregular ulcers [15]. The clinical similarities between herpetiform ulcers in BD and ulcers due to HSV infection suggest an etiologic role of HSV in BD and several studies have attempted to isolate HSV from the oral ulcers of patients with BD. HSV-1 DNA fragments have been detected by PCR [8] or *in situ* hybridization [7] in significant numbers in the peripheral blood leukocytes of patients with BD; however, viral DNA has not been detected in biopsy samples taken from oral ulcers, even in the presence of high anti-HSV-1 antibody concentrations in the peripheral blood of BD patients [8, 16]. The inability to detect viral DNA in tissue could be due to the viral DNA being present in small fragments rather than as an intact viral genome [8]. To further explore the role of HSV in the pathogenesis of BD, our group evaluated the presence of HSV DNA in saliva samples from 66 patients with the disease. The 289-bp band specific for HSV DNA was detected in DNA preparations from the saliva of 26 (39.4%) patients [9].

Although less common than oral lesions, patients with BD also often have genital lesions, which are characterized by ulcers. Clinically differentiating BD genital ulcers from HSV-induced ulcers (the most common type of genital ulcer in developed countries) is often difficult [17]. The clinical similarities suggest that HSV has a pathogenic role in the development of genital ulcers in BD. Moreover, HSV-1 DNA has been identified by PCR in biopsy samples obtained from genital ulcers of BD patients but not in biopsies from healthy controls [18].

Various cutaneous manifestations may be found in BD, including an erythema multiforme-like rash, which accounts for approximately 5% of BD skin lesions [19]. Conventional erythema multiforme is a hypersensitivity reaction associated with certain infections and medications. It can involve the oral, ocular, genital, upper respiratory, or pharyngeal mucosa [20]. HSV is the most common precipitating agent. HSV-associated erythema multiforme is usually limited to the oral mucosa, more specifically, the labial and buccal mucosa, nonattached gingivae, and vermillion lip [21]. Except for the occurrence of a prodromal episode of recurrent herpes infection in HSV-associated erythema multiforme, it is often difficult to distinguish this condition from the erythema multiforme-like lesions in BD. Because of this, many diagnostic criteria for BD do not include erythema multiforme-like rash as a disease-defining skin manifestation [22, 23]. Till now direct detection of HSV DNA from erythema multiforme-like lesions in BD has not been reported although the presence of HSV-1 and HSV-2 genomes in skin lesion from patients with BD was infrequently reported [24]. However, the clinical similarities between HSV-associated erythema multiforme and erythema multiforme-like lesions in BD are further evidence supporting an association between HSV and BD.

## 3. The Effect of HSV Infection on the Expression of Cell Adhesion Molecules in Cultured Human Dermal Microvascular Endothelial Cells

Cell-mediated immune responses to HSV have been implicated in the pathogenesis of various inflammatory diseases, including BD. HSV DNA and viral antigens are frequently detected in skin lesions of HSV-associated erythema multiforme [25], but it is unclear whether the pathologic changes in skin lesions are caused by the virus itself or by a secondary immunological reaction to a viral antigen. *In vitro* studies of cultured human dermal microvascular endothelial cells (HDMECs) have demonstrated that exposure to HSV-1 increased binding of T cells to HDMECs [26] and increased expression of cell adhesion molecules, such as CD54 (intercellular adhesion molecule-1 [ICAM-1]), vascular adhesion molecule 1 (VCAM-1), and E-selectin by HDMECs [26]. Similarly, incubation of HSV-infected peripheral blood mononuclear cells with uninfected HDMECs induced upregulation of CD54 and major histocompatibility complex class I molecules on HDMECs [27]. The binding of T cells to HDMECs was inhibited by anti-CD54, anti-interleukin (IL)-1*α*, or anti-tumour necrosis factor (TNF)-*α* antibody [26]. These *in vitro* data suggest that IL-1*α* or TNF-*α* produced by HSV-infected HDMEC may function in the immunopathogenesis of BD. Thalidomide is a TNF-*α* inhibitor that is effective in both BD patients [28] and a BD-like mouse model [29]. The TNF-*α* blockers infliximab and etanercept have both been shown to reduce serum concentrations of soluble ICAM-1 and E-selectin in patients with arthritis [30, 31]. As well, a synthesized pyridine compound derivative (SK94) downregulated E-selectin, VCAM-1, and ICAM-1 in the HSV-induced BD mouse model [32]. Together with TNF-*α* inhibitors, targeting HSV-induced cell adhesion molecules on endothelial cells may thus be a promising therapeutic strategy for BD.

## 4. Behçet's Disease-Like Symptoms Induced by HSV in ICR Mice

To further explore the role of HSV in the pathogenesis of BD, we developed an HSV-induced BD mouse model. After inoculation of the scratched earlobe of 258 ICR mice with HSV type 1, 77 (29.8%) mice exhibited BD-like syndrome, defined by the presence of two or more BD symptoms. These symptoms and their overall frequency were as follows: skin ulcers (57.1%); eye symptoms (39.0%); partial hair loss (33.8%); genital ulcers (19.5%); bullae (11.7%); arthritis (5.2%); gastrointestinal ulcer (5.2%); and tongue ulcers (3.9%). The ulcers, uveitis, and arthritis were clinically similar to those seen in patients with BD. Skin lesions stained with hematoxylin and eosin showed accumulations of perivascular inflammatory cells, and vasculitis was common in the intestinal, oral, ear lobe, and genital epithelial lesions [11]. More recently, ^18^F-fluorodeoxyglucose positron emission tomography showed the presence of symptomatic and asymptomatic knee joint inflammation in BD mice [33]. Although BD-like symptoms have been induced by inoculating the scratched earlobe of mice, direct injection of HSV in the perioral area, tongue, cornea, peritoneum, and footpad has not produced BD-like symptoms (unpublished data). HSV-induced BD-like symptoms are dependent on the species of mice: symptoms have been observed in 40%–50% of HSV-inoculated B10.BR, B10.RIII, and C57BL/6 mice but only 2% of C3H/He strain mice [34]. The function of HLA-G (Qa-2 in mice), which is immunosuppressive gene, was also decreased in HSV-induced BD mice [35]. The species-specificity and inherent abnormality of HLA molecules in this mice model are also similar to the presence of genetic predisposition in BD patients.

## 5. Expression of Th2 Cytokine Decreases the Development of and Improves Behçet's Disease-Like Symptoms Induced by Herpes Simplex Virus in Mice

Although BD-like symptoms have been induced by HSV inoculation of mice, viral infection alone is insufficient to explain the pathogenesis of BD, as no more than half of inoculated mice develop BD symptoms [11, 34]. To study the possible role of immunologic abnormalities in the development of BD-like symptoms induced by HSV inoculation of ICR mice, macrophages were depleted by intraperitoneal injection of liposome-encapsulated clodronate [36]. The incidence of BD-like symptoms was 28% in mice injected with HSV alone and 0% in mice that underwent macrophage depletion plus HSV inoculation. Macrophage depletion correlated with increased IL-4 expression in the mice spleens. When type 2 T helper cell (Th2) adjuvant ovalbumin (OVA)-alum was injected into mice with BD-like symptoms, cytokine IL-4 was upregulated and cutaneous symptoms improved. Adoptive transfer with splenocytes from OVA-alum-injected normal healthy mice into BD mice also resulted in improvement. These findings thereby suggest that upregulated Th2 cytokine expression can prevent or improve at least some BD-like symptoms. Nagafuchi et al. [37] also reported Th1 cells and the Th1-associated cytokines may play a detrimental role in the development of skin lesions in patients with BD. 

## 6. Learning from HSV-Infected Mice as a Model of Behçet's Disease

Most animal models of disease are not perfectly matched to their corresponding human disease, but they are often the most appropriate research tool. Furthermore, the complexity of BD makes it difficult to develop a single experimental animal model to understand the mechanisms responsible for all BD symptoms. Currently, the HSV-induced BD mouse model is the model that most closely mimics the symptoms, histological characteristics, immunological abnormalities, and responses to therapeutic modalities of patients with BD. This mouse model has been a useful tool for the study of BD pathogenesis and pharmacotherapy. Since the successful development of an HSV-induced BD mouse model was first reported in 1998 [11], over 20 papers using this model have been published. These papers have involved such topics as immunological abnormalities, including major histocompatibility (MHC) relevance [34, 35], Th1/Th2 balance [36], and CD4^+^CD25^+^Foxp3^+^ regulatory T cells [38]; therapeutic targets, such as TNF*α* [39], IL-6 [40], and IL-17 [40]; and conventional and potential therapies involving colchicines [41, 42], thalidomide [29], famciclovir [34], gemcitabine (2′,2′-difluorodeoxycytidine) [43], IL-4 DNA vector [44], and vitamin D3 [45]. 

## 7. Innate Immunity and Tripartite Motif-Containing Proteins in Viral Infection

Tripartite motif-containing (TRIM) proteins, a family of RING domain-containing E3 ligases, play diverse functions in cell biology and immunity [46]. Several TRIM proteins exhibit direct antiviral activity, by restricting various stages of the viral replication cycle [47]. Some TRIM proteins also regulate signalling of immune pathways mediated by pattern recognition receptors [48]. With recent elucidation of the role of TRIM proteins as viral restriction factors or regulators of the viral RNA/DNA sensing pathway and the inflammasome, the role of TRIM proteins as key molecules in antiviral immunity has become increasingly apparent [47, 48]. TRIM proteins contribute to the regulation of immune responses, including the production of type I interferons and proinflammatory cytokines such as interleukin-1*β*, and they have been reported to have pathogenic roles in numerous autoimmune diseases, including systemic lupus erythematosus, gout, type 2 diabetes, rheumatoid arthritis, and Crohn's disease [47, 49, 50].

 Because of the role of TRIM proteins in antiviral immunity, pattern recognition receptor signalling, inflammasome activation, and autoimmunity, it has recently been suggested that these proteins may contribute to the pathogenesis of BD. A single nucleotide polymorphisms study published in 2010 demonstrated that TRIM39 was independently associated with BD, suggesting that it may contribute to the pathogenesis of the disease [51]. TRIM39R (although not TRIM39B) was subsequently reported to be related to the regulation of the type I interferon response [52]. 

 TRIM19, which is also known as the promyelocytic leukaemia (PML) protein, organizes PML nuclear bodies, which function in antiviral immunity [53]. The genomes of HSV-1 and human cytomegalovirus are both subject to transcriptional repression by mechanisms involving PML and nuclear body components [54]; these are important innate antiviral defence mechanisms. TRIM19 enhances interferon-*γ*-mediated antiviral gene expression [55], and its depletion has led to increased replication of HSV-1 in previous studies [53, 54]. As HSV infection appears to play an important role in the pathogenesis of BD and TRIM19 functions in innate defence mechanisms against HSV, this suggests that TRIM19 may contribute to the development of BD.

 Several other TRIM proteins also possess antiviral properties and are being investigated as potential mediators of autoimmune or chronic inflammatory disorders [46–48]. However, studies regarding the relationship between TRIM proteins in BD are rare. Since TRIM proteins have the potential to function as a potent cellular detection mechanism and can allow cells to stimulate broad-spectrum immunity [56], they may be candidate molecules for connecting the innate immune system to the pathogenesis of BD. Therefore, further investigations to reveal the role of TRIM proteins in BD might aid in further elucidating the pathogenesis of this disease.

## 8. Therapeutic Efficacy of HSV-Targeted Treatments in BD Patients and the BD Mouse Model

There is currently no single curative therapeutic agent for BD. Present treatment of BD is empirical and focuses on symptom relief, not a complete cure. The treatment generally depends on the clinical manifestations of each patient and is thus primarily patient-specific [57–60]. Although various therapeutic agents have been used for BD, the treatment results vary widely between agents and individuals, and the results are far from optimal [60]. 

As HSV has been substantially implicated in the pathogenesis of BD [7–11], most strategies have been centred on treating the HSV infection itself or blocking the HSV-related immunologic pathways. Although treatment with acyclovir failed to reduce the frequency and severity of orogenital ulceration or other clinical manifestations in BD patients [61], treatment with famciclovir, a prodrug form of penciclovir that inhibits viral DNA polymerase, was effective in improving BD-like symptoms and preventing relapse in the mouse model of BD [34].

TNF-*α*, a representative proinflammatory cytokine, is primarily produced by T cells, polymorphonuclear cells, dendritic cells, and macrophages [62]. In macrophages, production of TNF-*α* can be induced by physical, chemical, and biologic stimuli, including ischemia, trauma, irradiation, tumour cells, complement, and various other cytokines [39]. The TNF-*α* signalling pathway can also be induced by bacterial and viral infections. As viral infection appears to be a triggering factor for BD, studies focusing on reducing or blocking TNF-*α* signalling have been performed. Injecting TNF-*α* small interfering RNA or anti-TNF-*α* blockades (infliximab or etanercept) inhibited TNF-*α* gene expression and improved symptoms in a BD mouse model [39]. Synthesized pyridine compound derivatives (SK94 and SK126) are also potential therapeutic candidates for BD, as they have been shown to downregulate cell adhesion molecules and TNF-*α*, and ameliorate symptoms in the BD mouse model [32].

Investigations continue to be conducted to more fully elucidate the pathogenesis of BD and to develop novel therapeutic agents. Peptides such as Hsp-65/60 are now being considered as possible new therapeutic targets for patients with BD [63]. Further investigations, including clinical trials in humans, are required to validate the effectiveness and safety of candidate agents. Additionally, more in-depth studies are required to investigate the role of immune reactions in BD, including not only adaptive responses but also innate immune reactions, such as those related to TRIM proteins. 

## 9. Conclusion

The exact pathogenesis of BD remains elusive, although research continues to increase our understanding of the complex pathogenetic mechanisms of the disease. Infectious agents, such as HSV and *Streptococcus sanguinis*, have long been postulated as possible environmental triggers of BD, and the role of HSV continues to be a main focus of BD research. Clinical observations and the detection of HSV DNA in saliva and genital ulcers of BD patients prompted us to investigate the effects of HSV in BD *in vitro* and *in vivo*. Establishing a BD mouse model using HSV inoculation facilitated our investigations of the pathogenesis of BD and, more importantly, the therapeutic efficacy of emerging medicines. As current treatment options for BD are limited and often unsatisfactory, it is hoped that recent research findings will lead to the development of successful therapeutic strategies for BD in the near future.
